# Supplementary material for: Synergistic and antagonistic impacts of suspended sediments and thermal stress on corals
Source: Nat Commun. 2019 May 28;10:2346. doi: 10.1038/s41467-019-10288-9 (PMC6538670; doi:10.1038/s41467-019-10288-9)
Supplement: Supplementary file 1 — Supplementary Information [file 41467_2019_10288_MOESM1_ESM.pdf]

Supplementary tables and figures accompanying paper

‘Synergistic and antagonistic impacts of suspended sediments and thermal stress on corals’

Rebecca Fisher[1,2]*, Pia Bessell-Browne[1,3], Ross Jones[1,2]

[1]Australian Institute of Marine Science (MO96), University of Western Australia, 35 Stirling Hwy, Crawley, Western Australia, 6009, Australia.

[2]Western Australian Marine Science Institute (MO95), University of Western Australia, 35 Stirling Hwy, Crawley, Western Australia, 6009, Australia.

[3]The Centre for Microscopy, Characterisation and Analysis (MO10), University of Western Australia, 35 Stirling Hwy, Crawley, Western Australia, 6009, Australia.

Corresponding author email: r.fisher@aims.gov.au

2 supplementary tables

5 supplementary figures

Supplementary Table 1 Predictor variables used in statistical models of mortality.

Parameter	Units	Range	Description
Bleached	Binary	0, 1	A factor variable indicating if there was evidence of thermal bleaching in the previous field survey (thermal bleaching score 2 or greater). This is an indicator of thermal stress.
Sediment cover	Binary	0, 1	A factor variable indicating if there was evidence of sediment on coral tissue in the previous field survey (sediment cover score 3 or greater). The sediment may be on coral tissue or on mucous sheet, and is an indicator of sediment stress.
SSC (14 d)	Log_{10} (NTU+1)	0.1–1.5	Suspended sediment loads as measured through nephelometric turbidity units (NTU). Values used were the worst case running 14 day mean NTU value observed in the time period preceding each photograph, summarised for each site. Running means were calculated as described in [1] as: $\bar{x}_T\ (t) = \frac{1}{N_T}\sum_{i=1}^{N_T} x_i\ (t)$, where NT is the number of samples in the T day mean. $\bar{x}_T$ is the mean calculated over the previous T days, $x_i(t)$ are the N_T data points up to and including time t.
Light (14 d)	$3\sqrt{1-(DLI/30)}$	0.3–0.9	A light stress index capturing proportional lost light, calculated following [2], as one minus the cube-root of the observed DLI (mol photons m^{-2} d^{-1}) relative to a fixed maximum (30 mol photons m^{-2} d^{-1}).). The transformation generates a metric of available light where 1 indicates no light at a given site, and zero indicates a theoretical maximum of 30 mol m^{-2} d^{-1}, which emphasises differences among low light values and dampens the influence of very high light values. DLI were calculated from 10 minute PAR readings following the methods and data cleaning procedures described in Jones, et al. [1]. Values used were the wort case 14 day running mean value observed in the time period preceding each photograph (as for SSC see above).
Sedimentation (60 d)	NA	0-1	Sedimentation index based on surface accumulated sediment as measured using an upward facing nephelometer (see [3]), summarised as the worst case 60 day running mean observed in the time period preceding each photograph (as for SSC above).
Temperature	°C	21.6–31.4	Mean daily temperature, summarised as the worst case observed in the time period preceding each photograph.
Depth	m	3.8–11.1	Median water height, summarized for each site.
Distance	km	0.19-32.8	Distance in Km to the nearest edge of the dredging footprints, summarized for each site.

Supplementary Table 2 Model fit statistics for predicting coral health.

	Fitted model	ΔAICc	ωAICc	edf
**a** Probability of any bleaching (bleaching incidence)				
	Taxa/group + Light × Temperature	0	1	26
**b** Probability of any mortality (mortality incidence)				
Branching	Bleached + Light × Bleached + Temperature × Bleached	0	0.49	18
	Light + Bleached + Temperature × Bleached	1.1	0.29	14
Massive	Sedimentation + Bleached + Temperature × Bleached	0	0.19	14
	Bleached + Sedimentation × Bleached + Temperature × Bleached	0.32	0.16	18
	Depth + Bleached + SSC × Bleached	0.43	0.16	14
	Sedimentation + Bleached	0.54	0.15	6
	Sedimentation + Temperature + Bleached	1.2	0.1	10
	Bleached + SSC × Depth	1.9	0.07	26
**c** Proportional live tissue loss				
Branching	Bleached	0	0.18	2
	Temperature + Bleached	0.31	0.15	6
	Null	1.9	0.07	1
Massive	Sedimentation + Temperature + Bleached	0	0.22	10
	Temperature + Bleached + Light × Bleached	0.35	0.19	14
	Light + Temperature + Bleached	0.80	0.15	10
	Light + Bleached	0.85	0.15	6
	Sedimentation + Temperature + Bleached	0	0.22	10
	Temperature + Bleached + Light × Bleached	0.35	0.19	14
	Light + Temperature + Bleached	0.80	0.15	10

Included are all generalised additive mixed models (GAMM) within 2 AICc for the relationship between various environmental pressure metrics and stress indicators, and the probability of observing bleaching (a), the probability of observing any partial mortality (mortality incidence, b) and the proportional coral loss given a partial mortality incidence occurred (c). Shown are ΔAkaike Information Criterion (AICc), AICc model weights (ωAICc), total estimated df.

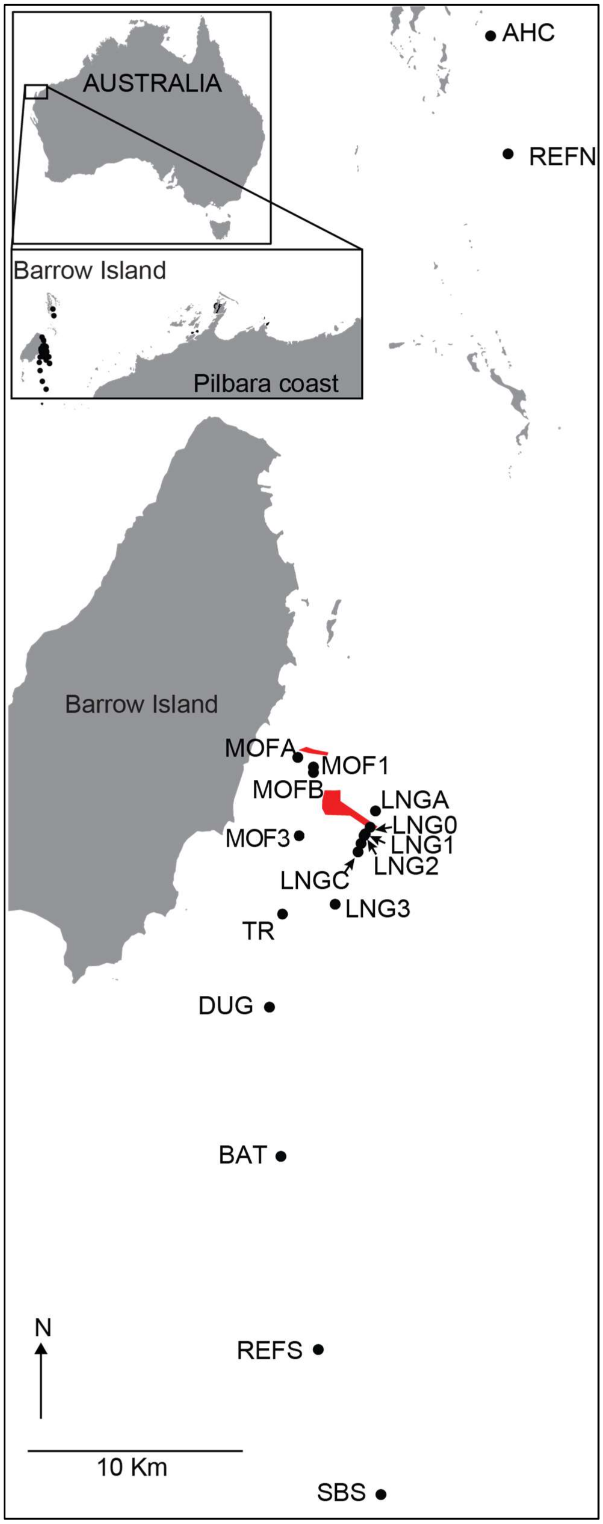

Supplementary Figure 1 Sampling sites and location of Barrow Island in NW of Western Australia off the Pilbara coast. Labels indicate the 17 water quality and coral health monitoring sites relative to the primary excavation areas (a materials offloading facility [MOF sites] and turning basin and tanker access channel [LNG sites], shown in red).

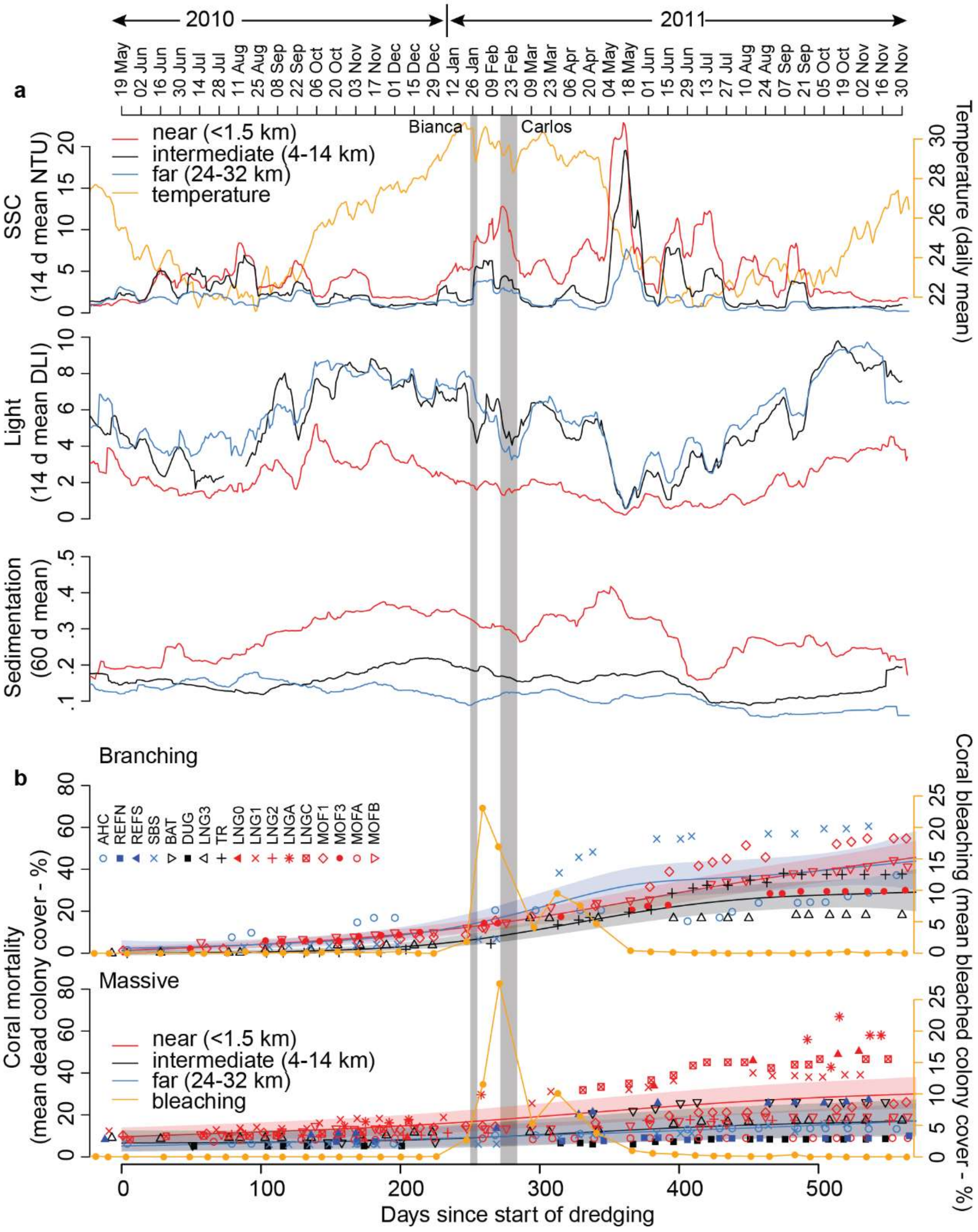

Supplementary Figure 2 Time series of water quality and coral health monitoring data collected at Barrow Island. Data were summarized for plotting purposes into three groups, those near (red), intermediate (black) and far (blue) from dredging. The impact of dredging on water quality conditions is evident for all three metrics of direct (SSC) and indirect (light and sedimentation index) dredging related stress (Supplementary Table 1) across the entire 530 days of dredging (**a**). Thermal stress was widespread across the region during early 2011 (yellow line, a), although appeared to dip in response to both cyclone Bianca and cyclone Carlos (vertical grey bands). Coral health data showed a gradual increase in the mean proportional mortality (**b**), with marked elevations in mortality following peak mean coverage of bleaching.

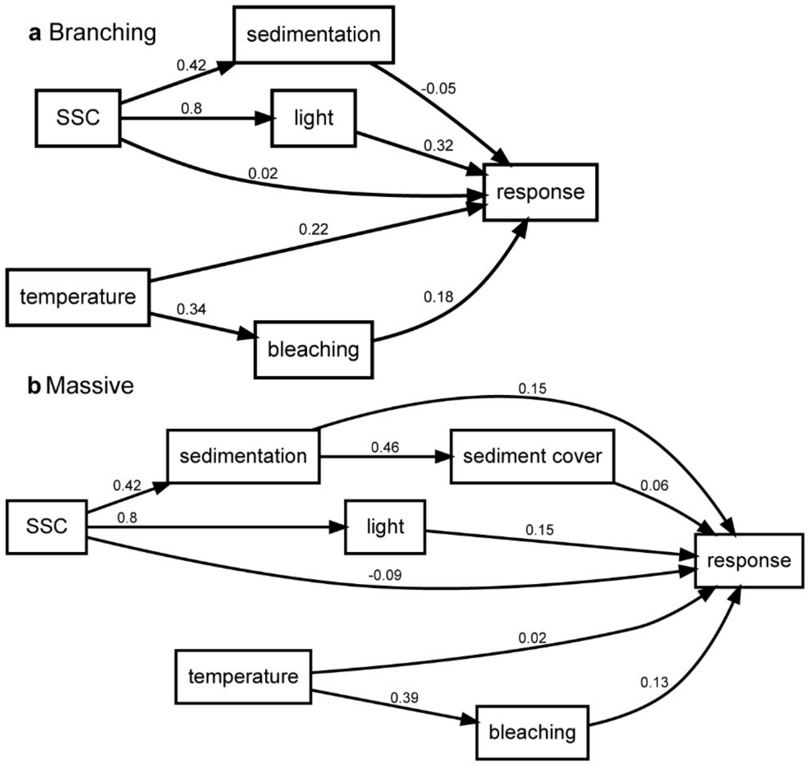

Supplementary Figure 3. Path diagrams used in structured equation models. For both branching (**a**) and massive (**b**) corals the response variable modelled was the logit of the proportional mortality of corals between each image (live cover at time 1 – live cover at time 2 (see 'Coral health monitoring'). An a priori full model was developed for each coral group. For both branching and massive corals the suspended sediment concentrations (SSC) could directly cause partial mortality, or could cause reductions in light availability and increased sedimentation. Reduced light availability could directly cause partial mortality, also for both coral morphology types. Sedimentation could cause partial mortality directly, or via sediment cover, which may subsequently result in partial mortality. As branching corals were never observed with sediment covering their surfaces, this link was not included in the branching coral mortality path diagram.

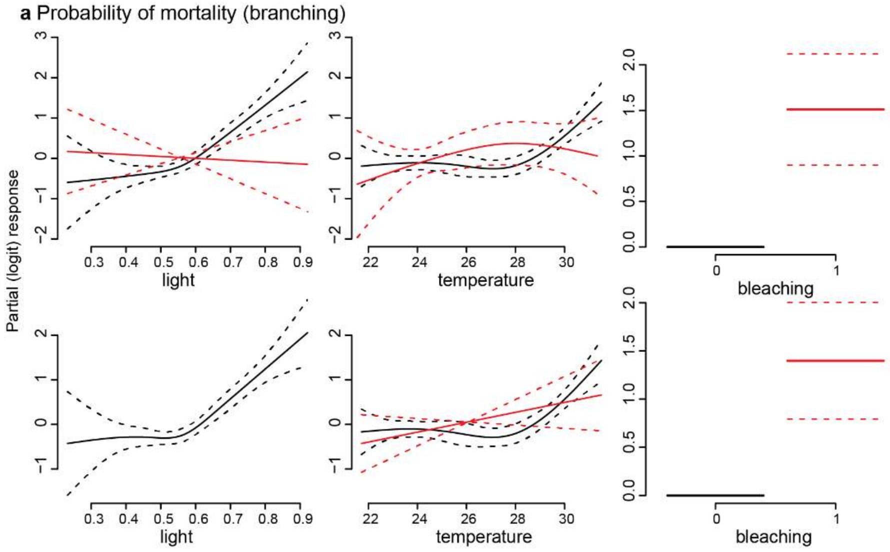

Supplementary Figure 4a. Best fit models for the probability of branching coral partial mortality. Partial plots for all generalised additive mixed models (GAMM) within 2 AICc (see Supplementary Table 2) are shown for each of the included environmental predictors. Confidence bounds represent 2 × SE.

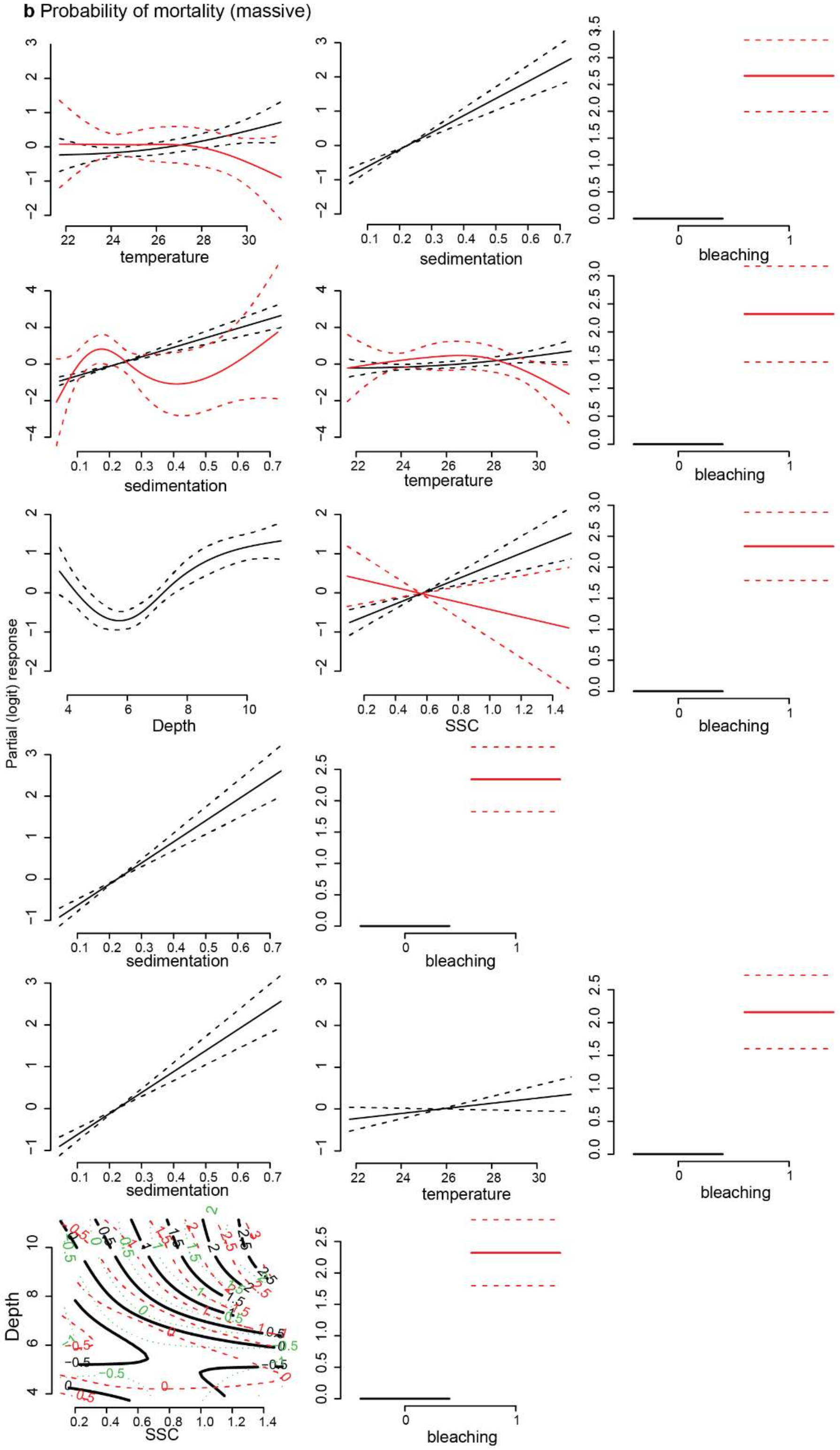

Supplementary Figure 4b. Best fit models for the probability of massive coral partial mortality. Partial plots for all generalised additive mixed models (GAMM) within 2 AICc (see Supplementary Table 2) are shown for each of the included environmental predictors. Confidence bounds represent 2 × SE.

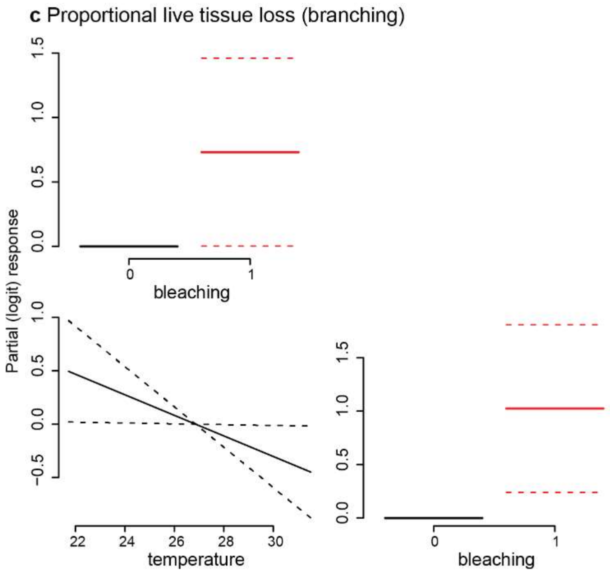

Supplementary Figure 4c. Best fit models for proportional branching colony loss. Partial plots for all generalised additive mixed models (GAMM) within 2 AICc (see Supplementary Table 2) are shown for each of the included environmental predictors. Confidence bounds represent 2 × SE.

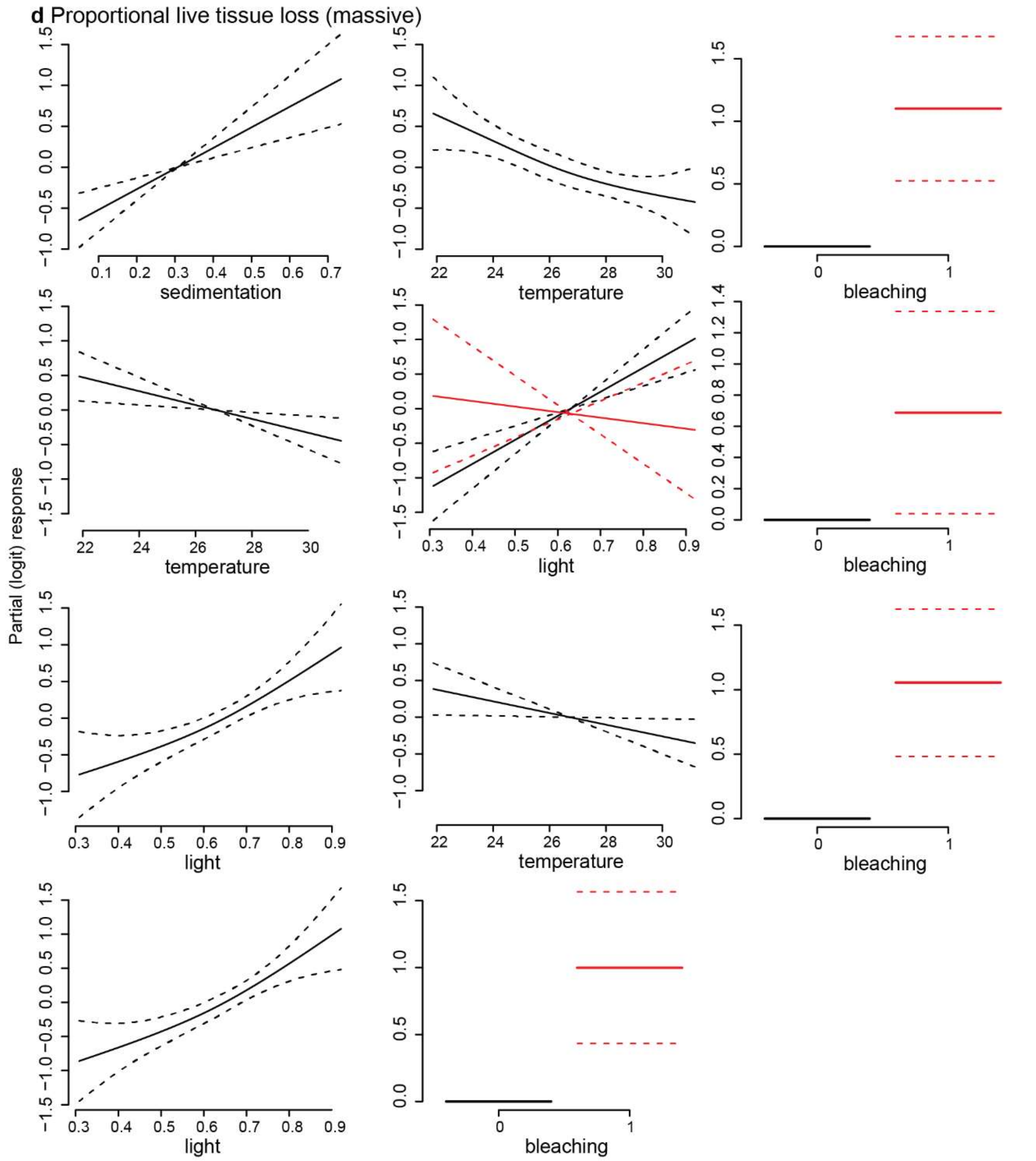

Supplementary Figure 4d. Best fit models for proportional massive colony loss. Partial plots for all generalised additive mixed models (GAMM) within 2 AICc (see Supplementary Table 2) are shown for each of the included environmental predictors. Confidence bounds represent 2 × SE.

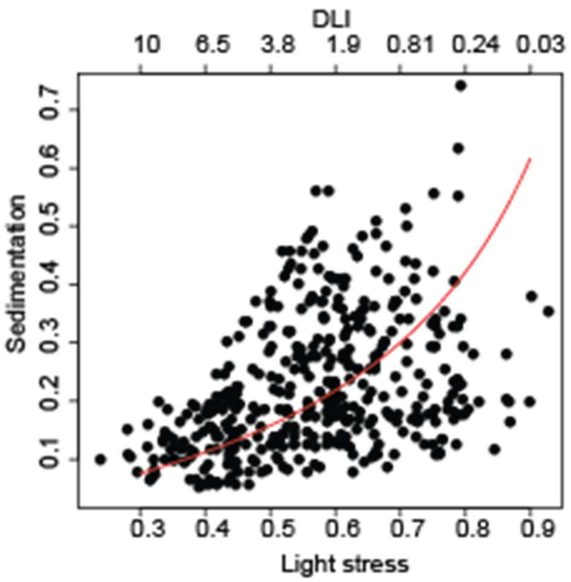

Supplementary Figure 5. Relationship between light stress and sedimentation at Barrow Island. Data are the site level mean worst case 14 d running mean light stress (see Supplementary Table 1) and 60 d running mean sedimentation (see Supplementary Table 1) between each field survey, as used in the modelling to predict coral mortality. Red lines shows the Generalised Additive Mixed Model relationship as fit using gamm4, with logit sedimentation used as the response variable.

Supplementary References

1 Jones, R., Fisher, R., Stark, C. & Ridd, P. Temporal patterns in water quality from dredging in tropical environments. *PlosOne* **10**, e0137112. doi:0137110.0131371/journal.pone.0137112 (2015).

2 Fisher, R., Walshe, T., Bessell-Browne, P., Jones, R. & Trenkel, V. Accounting for environmental uncertainty in the management of dredging impacts using probabilistic dose-response relationships and thresholds. *Journal of Applied Ecology* **55**, 415-425, doi:10.1111/1365-2664.12936 (2018).

3 Ridd, P. *et al.* Measurement of sediment deposition rates using an optical backscatter sensor. *Estuarine Coastal and Shelf Science* **52**, 155-163, doi:DOI 10.1006/ecss.2000.0635 (2001).
